# Bone marrow-derived Ly6C^−^ macrophages promote ischemia-induced chronic kidney disease

**DOI:** 10.1038/s41419-019-1531-3

**Published:** 2019-03-29

**Authors:** Qian Yang, Yuxi Wang, Guangchang Pei, Xuan Deng, Hongyang Jiang, Jianliang Wu, Cheng Zhou, Yi Guo, Ying Yao, Rui Zeng, Gang Xu

**Affiliations:** 1 Division of Nephrology, Tongji Hospital, Tongji Medical College, Huazhong University of Science and Technology, No. 1095 Jiefang Ave, Wuhan, 430030 Hubei China; 2 Division of Urology, Tongji Hospital, Tongji Medical College, Huazhong University of Science and Technology, No. 1095 Jiefang Ave, Wuhan, 430030 Hubei China

## Abstract

Macrophages play an important role in renal injury and repair after acute kidney injury (AKI) and the subsequent chronic kidney disease (CKD) that often results. However, as macrophages have a high degree of plasticity and heterogeneity, the function(s) of macrophage subtypes in AKI-to-CKD progression are not fully understood. Here, we focused on Ly6C^−^ macrophages, which are derived from the embryonic yolk sac and post-development become resident in the kidneys. We found that C–C chemokine receptor type 2 (CCR2) deficiency, which blocks the migration of Ly6C^+^ macrophages from the bone marrow to the sites of injury, alleviated ischemia-induced AKI in mice. Unexpectedly, though, CCR2 deficiency worsened the subsequent renal fibrosis, which was marked by notable intra-renal infiltration of Ly6C^−^ macrophages. These Ly6C^−^ macrophages were greater in number in both the acute and chronic phases after ischemia reperfusion (I/R) in kidneys of wild type (WT) mice, and we showed them to be derived from the bone marrow by bone marrow chimerism. Clodronate Liposomes (CLs)-mediated depletion of renal Ly6C^−^ macrophages in CCR2^−^^/−^ mice or in WT mice after I/R alleviated the renal injury and fibrosis. On the contrary, adoptive transfer of Ly6C^−^ macrophages from injured kidneys of WT mice into immune-deficient mice was sufficient to induce renal injury and fibrosis. Transcriptome sequencing of Ly6C^−^ macrophages from injured kidneys revealed that they secreted various cytokines and growth factors, which were associated with the transdifferentiation of fibroblasts into myofibroblasts. This transdifferentiation effect was further supported by in vitro studies showing that Ly6C^−^ macrophages induced the secretion of extracellular matrix proteins from co-cultured fibroblasts. In conclusion, the presence of bone marrow-derived Ly6C^−^ macrophages after ischemia induces AKI and worsens subsequent CKD.

## Introduction

Acute kidney injury (AKI), defined as the rapid deterioration of renal function, is a common clinical status resulting from various pathogenic conditions such as renal ischemia or toxic insults and has been recognized as a life-threatening pathology^1^. Recent clinical studies have demonstrated that even extremely mild AKI with complete recovery can greatly increase the risk of subsequent progression to chronic kidney disease (CKD) and end-stage renal disease (ESRD)^2,3^. This “AKI-to-CKD progression” has led to striking socioeconomic and public health outcomes for all countries and highlights the urgent need for novel therapeutic strategies to prevent this disease course^4^.

Renal fibrosis is the main pathological feature of AKI-to-CKD progression^5^, characterized as the persistent activation of myofibroblasts and multiple inflammatory cells^6^, especially macrophages^7^. Accumulating evidence has suggested that persistent macrophage infiltration is regarded as an important driving force of CKD progressing to ESRD^8–10^. However, macrophages are highly heterogeneous and diverse in their origin and function^11^. Different subpopulations play various roles at different stages in the course of renal disease^12^. Indeed, the functions of diverse macrophage subtypes in inflammation and tissue remodeling are not fully understood.

Research over the past decades has focused on identifying and characterizing macrophage subpopulations that regulate tissue injury and repair^13^. It is generally suggested that macrophages are divided into M1-like (i.e., pro-inflammatory) macrophages and M2-like (i.e., anti-inflammatory) macrophages^14^, but such a simple catagorization ignores the complexity of the micro-environment of macrophages and the functional crossover between M1-like and M2-like macrophages, leading to confusion regarding the role of macrophages in chronic kidney disease^15,16^. For example, M2-like macrophages have been shown to contribute to repair in damaged kidneys^17^, but other studies have implicated M2-like macrophages in the promotion of fibrosis^18^. The multiple functions of M2-like macrophages under different pathological conditions greatly limit the methods of intervention. Thus, further research is required to define more precisely the phenotypes of macrophages and their roles in the pathology of CKD.

Ly6C, a 14-kD protein, has been identified as an antigen expressed on 50% of bone marrow cells and a small population of T lymphocytes in the periphery^19^. Recent studies have shown that macrophage subpopulations are distinguished by differential Ly6C expression, which has been recognized as a superior marker to identify monocyte and macrophage subpopulations^14,20,21^. It is reported that Ly6C^+^ macrophages are thought to be derived from the peripheral circulation and have a pro-inflammatory function^22–25^, while Ly6C^−^ macrophages, generally regarded as tissue-resident macrophages, are considered to be derived from the embryonic yolk sac^13,14^. Accumulating studies have shown that tissue-resident macrophages play an important role in maintaining immune homeostasis and tissue regeneration in different organs such as the liver^26^, lung^27^, brain^28^, and gastrointestinal tract^29^. It has also been reported that the transfer of Ly6C^−^ monocytes rescues CD169(+) cell-depleted mice from lethal renal injury^30^, whereas other studies have reported that Ly6C^−^ macrophage subpopulation has a pro-fibrotic phenotype in unilateral ureteric obstruction (UUO) and in I/R-induced CKD mouse models^22,31^. As the existing views on the function of Ly6C^−^ macrophages in kidney diseases are controversial, the role of Ly6C^−^ macrophages in the progression of AKI to CKD was explored in this study. We focused on defining macrophage populations based on Ly6C expression, clarifying the origin of Ly6C^−^ macrophages, and exploring the function and mechanism of Ly6C^−^ macrophages in a murine model of ischemia-induced AKI-to-CKD progression.

## Materials and Methods

### Animal experiments

C57BL/6-CCR2^−/−^ and C57BL/6-Tg (CAG-EGFP) mice were purchased from Jackson Laboratory, USA. The control for C57BL/6-CCR2^−/^^−^ mice were wild type littermates from heterozygous parents. NOD-Prkdc^em26Cd52^Il2rg^em26Cd22^/Nju (NCG) mice, the genetic background of which are NOD/ShiLtJNju, were purchased from Nanjing Model Animal Research Center (Nanjing, China). All mice were maintained and housed in accordance with the Experimental Animal Ethics Committee of Huazhong University of Science and Technology. Mice were anaesthetized with 1% sodium pentobarbital solution (0.01 mL/g body weight, Sigma, USA) by intraperitoneal injection. The left renal pedicle was clamped with an atraumatic vascular clip for 30 min (Roboz Surgical Instrument Co, Germany) through a flank incision and the left kidney turned black subsequent to clamping. Clamps were removed after 30 min. Body temperature was controlled at 36.8–37.2 °C throughout the procedure (FHC, USA). Sham animals were subjected to a similar surgical procedure without clamping the left kidney pedicle.

### RNA extraction and Q-PCR

Total RNA was extracted from renal tissues with Trizol reagent according to the manufacturer’s instructions (Invitrogen, USA) and cDNA was synthesized using the reverse transcription system-kit (Takara, Japan). Quantitative PCR was performed using the SYBR master-mix (Takara, Japan) on the Roche light 480II. Relative mRNA expression levels were calculated using the 2−ΔΔCt method and were normalized to the expression levels of GAPDH. The following primers were used: GAPDH, forward 5′-TTGATGGCAACAATCTCCAC-3′, reverse 5′- CGTCCCGTAGACAAAATGGT-3′, F4/80, forward 5′- CTTTGGCTATGGGCTTCCAGTC-3′, reverse 5′-GCAAGGAGGACAGAGTTTATCGTG-3′, α-SMA, forward 5′- GTCCCAGACATCAGGGAGTAA-3′, reverse 5′-TCGGATACTTCAGCGTCAGGA-3′, Fibronectin, forward 5′- GCTCAGCAAATCGTGCAGC-3′, reverse 5′- CTAGGTAGGTCCGTTCCCACT-3′, Collagen I, forward 5′-ATGGATTCCCGTTCGAGTACG-3′, reverse 5′- TCAGCTGGATAGCGACATCG-3′. CSF1, forward 5′- GTGTCAGAACACTGTAGCCAC-3′, reverse 5′- TCAAAGGCAATCTGGCATGAAG-3′, CSF2, forward 5′- GGCCTTGGAAGCATGTAGAGG-3′, reverse 5′- GGAGAACTCGTTAGAGACGACTT-3′, IL34, forward 5′- TTGCTGTAAACAAAGCCCCAT-3′, reverse 5′- CCGAGACAAAGGGTACACATTT-3′.

### Western blot

Kidney tissues were stored at −80 °C immediately after mice were killed. Tissue samples were homogenized in RIPA lysis buffer containing a protease inhibitor cocktail and phosphatase inhibitor cocktail (Servicebio, China). Equal amounts of proteins (40 μg) were loaded on an 10% sodium dodecylsulfate-polyacrylamide gel. The gel was transferred onto PVDF membranes (Roche, Switzerland). The membrane was blocked with 5% skimmed milk in TBST for 1 h at room temperature and incubated overnight at 4 °C with the following primary antibodies: α-SMA (1:2000, Abcam, UK), PDGFR-β (1:1000, Abcam, UK). The membranes were incubated with HRP-conjugated secondary antibodies and were visualized by enhanced chemiluminescence (ECL, BioRad, USA). Mouse monoclonal anti-GAPDH Ab (1:4000, Abbkine, China) was used as loading control. The signal intensity of the targeted band was quantified using Image J (NIH, USA).

### Renal histopathology, immunofluorescence, and fibrosis quantification

Kidneys were fixed in 4% neutral buffered formalin, embedded in paraffin, sectioned (4 μm). PAS staining was used to evaluate kidney pathological injury. Tubulointerstitial damage was assessed with quantification of damaged renal tubules. Renal tubular injury was defined as brush border loss, tubular dilation, cast formation, or tubular atrophy. Masson Blue and Sirius Red staining were carried out to estimate the extent of tubular-interstitial fibrosis.

For immunohistochemistry staining (IF), antigen retrieval was performed using citrate solution after deparaffinization and rehydration. The sections were blocked with 5% goat serum and then incubated with the following primary antibodies at 4 °C overnight: α-SMA (1:100, Abcam, UK), PDGFR-β (1:1000, Abcam, UK), Ki-67 (1:200, Abcam, UK). Cryostat-cut mouse kidney sections were stained for the presence of macrophages, using F4/80 (1:100; Invitrogen, USA), GFP (1:100, Abcam, UK), α-SMA (1:100, Abcam, UK) and further developed with fluorescent labeled secondary antibodies for IF. Nuclei were stained with DAPI.

We used the fibrosis-related biomarkers α-SMA and PDGFR-β for immunofluorescence detection. Meanwhile, collagen fibers were stained using Masson Dye and Sirius Red Dye to further determine the degree of fibrosis. We used the Image-Pro Plus 6.0 software to quantify the positive area.

### Flow cytometry

We prepared and stained single-cell suspension from kidneys according to description in a previous report^32^. Mice were perfused with cold PBS to remove leukocytes in the circulating blood. Following antibodies were used from BioLegend for FACS analysis: APC-conjugated anti–mouse/human CD11b Ab; PE/Cy7-conjugated anti–mouse Ly6C Ab; APC/PE-conjugated anti–mouse F4/80 Ab; FITC–conjugated anti–mouse CD45 Ab (clone 30-F11); PercpCy5.5-conjugated anti–mouse CX3CR1 Ab, and PE conjugated anti-mouse α-SMA Ab from R&D, USA.

### Adoptive transfer

We isolated Ly6C^−^ Macrophages from B6 mice at day 5 after I/R, then transferred them (5 × 10^4^) into NCG mice by renal subcapsular injection. Renal subcapsular injection was successfully accomplished according to the previously published protocol^33^. Mice were killed 5 days later. Ly6C^−^ macrophages were isolated using flow cytometry.

### Bone marrow transplantation

CD45.1^+^ C57BL/6 recipient mice were lethally irradiated (7.5 Gy) in RS2000 (Rad Source, USA) and 1 × 10^7^ bone marrow cells from C57BL/6-Tg (CAG-EGFP) mice were adoptively transfer via tail vein injection.

### Transcriptome sequencing and bioinformatics analysis

Total RNA was obtained from Ly6C^−^^/+^ and control macrophages using Trizol reagent (Invitrogen, USA). In brief, the total RNAs were subjected to cDNA

synthesis, fragmentation, adapter ligation, and PCR amplification. Sequencing was performed on Illumina HiSeq platform. The sequencing reads were further processed with determination of quality using the SOAPnuke tool. We mapped clean reads to mouse mm9 genome using HISAT (Hierarchical Indexing for Spliced Alignment of Transcripts). The fragments per kilobase million (FPKM) and DEGs were obtained using RSEM and DEGseq software, respectively (Fold Change ≥2 and Adjusted *P* value ≤0.001). R package was used for generation of GO and Kegg analysis.

### Cell culture and treatment

Ly6C^−^ macrophages were isolated from kidneys at day 5 after I/R using flow cytometry. Then we co-cultured Ly6C^−^ macrophages and mouse fibroblasts (NIH3T3) in transwell chambers for 72 h. Then we lysed NIH 3T3 to collect total RNA.

### Statistics

All results are mean±SD from at least three separate experiments. Either an unpaired two-tailed test (data with normal distribution) or the Mann–Whitney *U* test (data with nonnormal distribution) was used to compare 2 groups by GraphPad Prism 5.0. The statistical significance is expressed as follows: *P < 0.05; **P < 0.01; ***P < 0.001; and n.s., not significant.

## Results

### CCR2 deficiency alleviates AKI while aggravating renal fibrosis

**Fig. 1:**
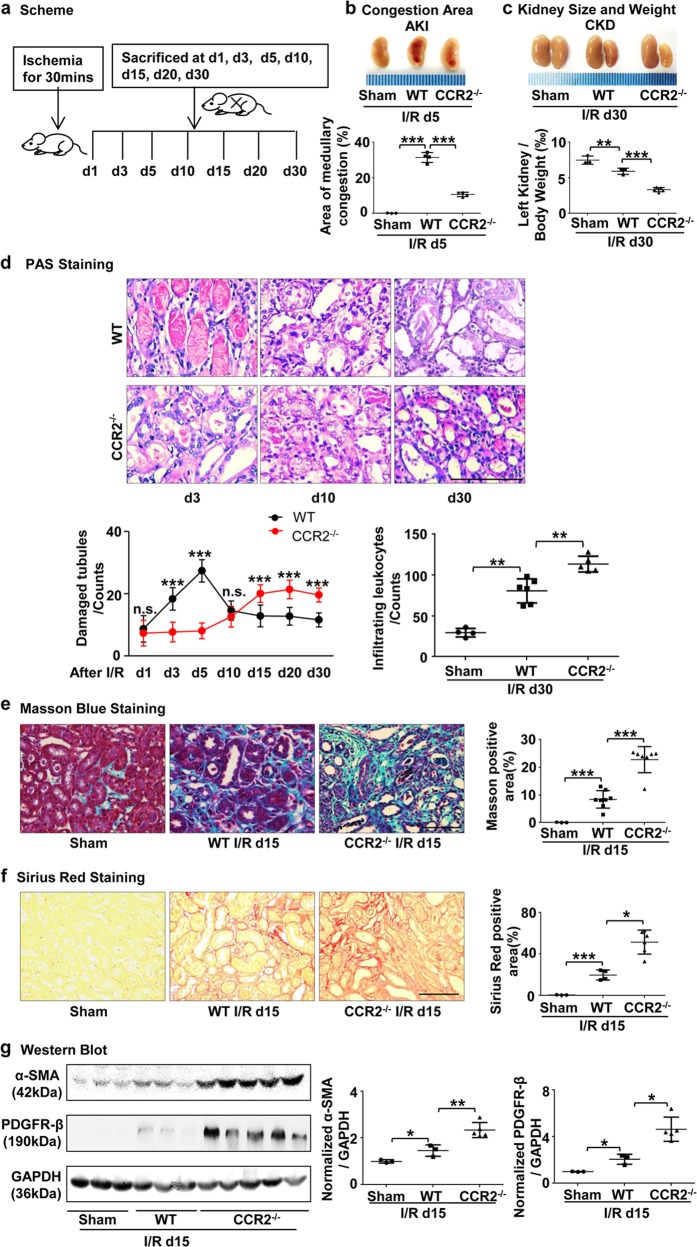
**CCR2 deficiency alleviates acute kidney injury, but aggravates renal fibrosis after I/R.** **a** Scheme: time-related comparison after I/R for AKI and CKD. **b**, **c** Representative kidneys from CCR2^–/–^ and WT mice and quantitative analyses for the area of medullary congestion (**b**) and kidney weight after I/R (**c**). **d** Representative photomicrographs of Periodic Acid-Schiff staining after I/R injury. Graphs indicate the tubular damage and infiltrating leukocytes after I/R. **e**, **f** Representative photomicrographs (left) of collagen staining by Masson blue (**e**) and Sirius red (**f**) at day 15 after I/R in the WT and CCR2^–/–^ kidneys. Corresponding graphs (right) indicate percentage of Masson blue and Sirius red. **g** Western blots of α-SMA and PDGFR-β at day 15 after I/R in the WT and CCR2^–/–^ kidneys. Quantitation of α-SMA and PDGFR-β relative to GAPDH. Scale bars, 100 μm. *N* = 3–6/group. *P < 0.05, **P < 0.01, ***P < 0.001. Values were means±SD

Due to the heterogeneity and plasticity of macrophages, the phenotype and function of macrophages change dramatically during the development of AKI to CKD. To determine the function of Ly6C^−^ macrophages, we used CCR2^−/−^ mice, which exhibit defective Ly6C^+^ monocyte recruitment from the bone marrow to inflammatory foci, and compared them to WT mice for AKI and subsequent CKD after I/R (Fig. 1a). We induced AKI and subsequent CKD by using unilateral ischemia-reperfusion injury (I/R) without nephrectomy, which has been identified as a very robust model in mice for induction of an AKI-CKD transition^34^. Consistent with previous reports, the area of medullary congestion was smaller in CCR2^−^^/−^ kidneys at day 5 after I/R compared to WT mice, indicating an alleviation of renal injury (Fig. 1b). However, at day 30, a time point at which CKD is present after I/R, kidney weight and size were strikingly smaller in CCR2^−/−^ mice compared with WT mice (Fig. 1c). Further, less injured tubules were observed in CCR2^−/−^ mice during the AKI phase, but there were a greater degree of atrophied tubules and leukocyte infiltration in the interstitium of the kidneys in the KO mice compared to WT during the chronic phase after I/R (Fig. 1d). Masson Blue (Fig. 1e) and Sirius Red (Fig. 1f) staining for collagen deposition showed aggravation of renal fibrosis in CCR2^−/−^ mice at day 15 after I/R compared to WT mice. By Western blotting we found that the protein expression levels of α-SMA and PDGFR-β, two markers of fibrosis, were greater in CCR2^−/−^ mice at day 15 after I/R vs WT (Fig. 1g). In addition, complement C5a receptor 1 (C5ar1) and complement C5a receptor 2 (C5ar2), which have recently been identified to be linked to AKI-CKD progression^35^, were upregulated in CCR2^−/−^ kidneys at day 30 after I/R compared to WT mice (Supplementary Fig. 1). Together, these data indicate that deficiency of CCR2, a chemokine receptor controlling the egress of inflammatory Ly6C^+^ monocytes from the bone marrow, alleviated AKI but worsened AKI-induced CKD.

### Ly6C^−^ macrophage increase occurs during CKD

**Fig. 2:**
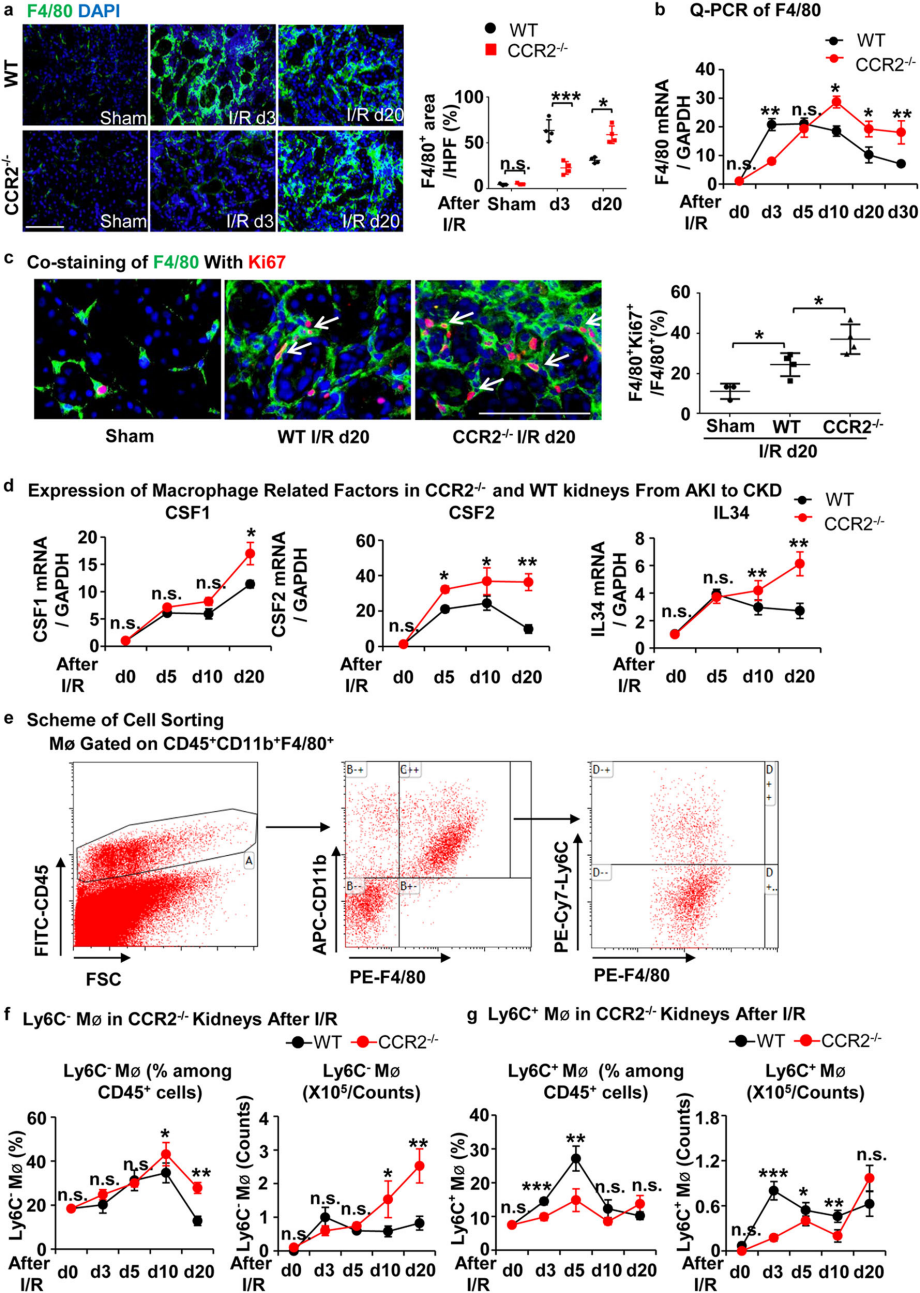
**In situ replenishment of Ly6C**^**−**^**macrophages compensates for CCR2 deficiency-induced macrophage sparsity in CCR2**^**−/−**^**kidneys during the chronic phase after I/R injury.** **a** Representative images (left) and quantitative analyses (right) for immunofluorescence labeled F4/80 (green) in the WT and CCR2^−/−^ kidneys at d3 and d20 after I/R. **b** Q-PCR for F4/80 mRNA level during AKI to CKD progression. **c** Representative images (left) and double-positive analyses (right) for immunofluorescence-labeled F4/80 (green) and Ki67(red) in the WT and CCR2^−/−^ kidneys at d20 after I/R. **d** Q-PCR for levels of macrophage growth factors CSF1, CSF2, and IL34 in CCR2^−/−^ and WT kidneys from AKI to CKD. **e** Scheme. cell-sorting approach. **f** The percentage (left) and number (right) of Ly6C^−^ macrophage analyses by flow cytometry from the whole kidneys in the WT and CCR2^−/−^ mice. **g** The percentage (left) and number (right) of Ly6C^+^ macrophage analyses by flow cytometry from the whole kidneys in the WT and CCR2^−/−^ mice. Scale bars, 100 μm. *N* = 4–6/group. *P < 0.05, **P < 0.01, ***P < 0.001. Values were means±SD. Mø, macrophages

We observed a large number of infiltrated leukocytes in the kidneys of CCR2^−/−^ mice during the CKD phase as shown in Fig. 1d, and thus we hypothesized that other subpopulations of macrophages besides Ly6C^+^ macrophages compensate for the loss of these inflammatory macrophages. To test this hypothesis, we conducted immunofluorescence staining and Q-PCR of F4/80, a general marker of macrophages. As expected, the number of intra-renal macrophages was lower during AKI in CCR2^−/−^ mice vs WT, but were replenished at the subsequent CKD phase (Fig. 2a, b). Co-staining for expression of Ki67 and F4/80 showed that the proliferation of macrophages in CCR2^−/−^ mice was greater than that of WT mice at day 20 after I/R (Fig. 2c). Further, the expression of macrophage growth factors, CSF1, CSF2, and IL34, were higher at the CKD phase in CCR2^−/−^ kidneys compared to WT (Fig. 2d), which suggests a formation of a pro-proliferative micro-environment for macrophages in CCR2^−/−^ kidneys after I/R injury. By flow cytometry we found that a greater number of intra-renal Ly6C^−^ macrophages (CD45^+^CD11b^+^F4/80^+^Ly6C^−^) occurred in the kidneys from CCR2^−^^/−^ mice compared to WT (Fig. 2e, f), while Ly6C^+^ macrophages showed no significant difference in the CKD phase compared to WT mice (Fig. 2g). Our data demonstrates a greater degree of Ly6C^−^ macrophages occurs in the kidneys of CCR2^−/−^ mice at the CKD phase after I/R compared to WT mice.

### Ly6C^−^ macrophages are the dominant population in I/R-injured kidneys and are derived from the bone marrow

**Fig. 3:**
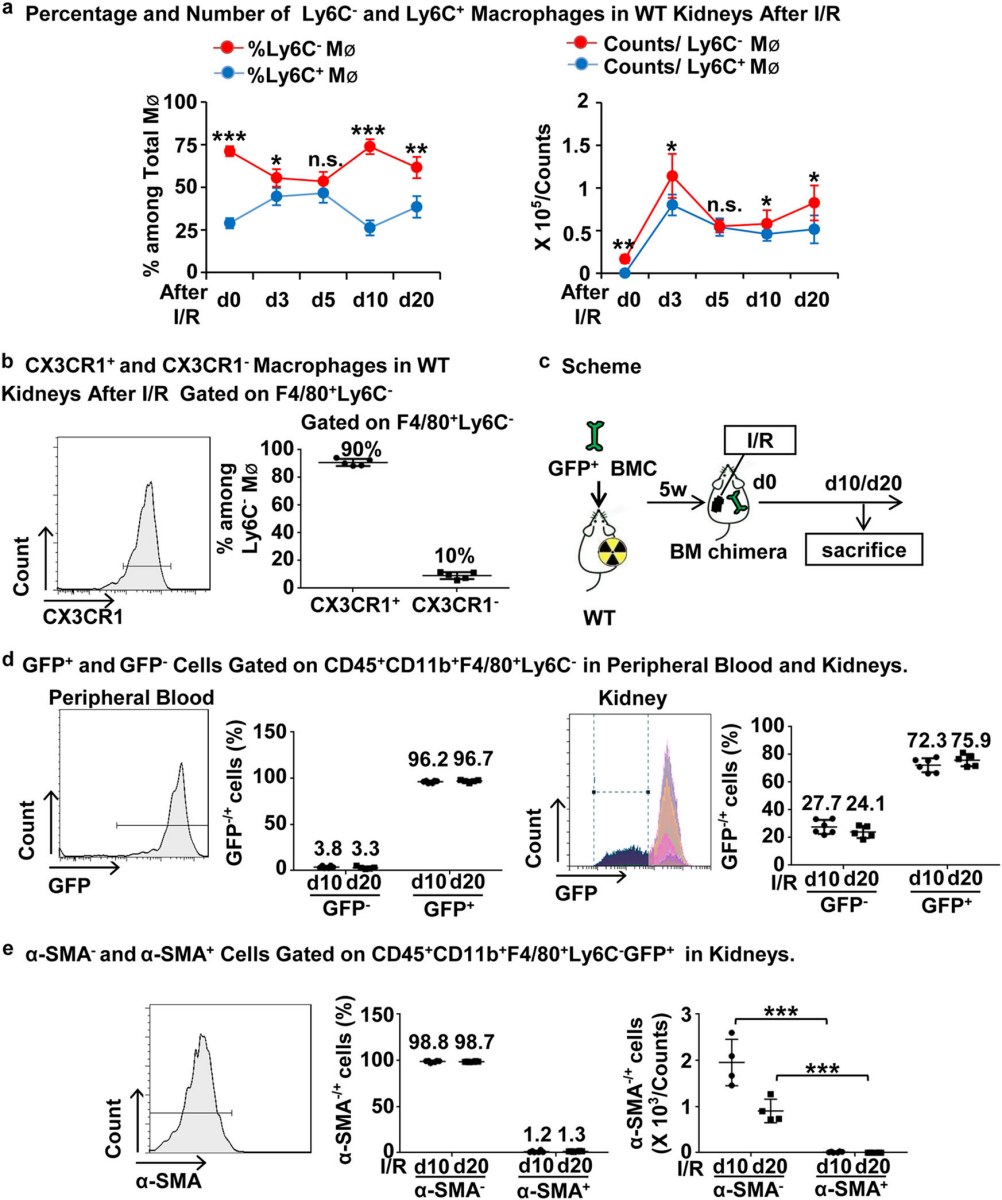
**Ly6C**^−^**macrophages, which are the dominant macrophages in WT kidneys in CKD after I/R injury, are mostly CX3CR1**^**+**^**, derived from bone marrow and incapable of differentiating into α-SMA**^**+**^**cells.** **a** The percentage (left) and number (right) of Ly6C^−^ and Ly6C^+^ macrophage analyses by flow cytometry from whole kidneys in WT mice. **b** The analyses by flow cytometry of percentage of CX3CR1^+^ and CX3CR1^−^ macrophages in WT kidneys at d7 after I/R gated on F4/80^+^Ly6C^−^. Graphs and representative plots. **c** Scheme of bone marrow chimera. **d** The percentage of GFP^−^ and GFP^+^ cells among Ly6C^−^ macrophage analyses by flow cytometry from the peripheral blood (left) and kidneys (right) of bone marrow chimeric mice at d10/d20 after I/R. Graphs and representative plots. **e** The analyses by flow cytometry of the percentage (middle) and number (right) of α-SMA^+^ and α-SMA^−^ cells among Ly6C^−^GFP^+^ macrophages from kidneys of bone marrow chimeric mice at d10/20 after I/R. Graphs and representative plots. Scale bars,100 μm. *N* = 4–6/group. n.s. *P* > 0.05, *P < 0.05, **P < 0.01, ***P < 0.001. Values were means±SD. Mø, macrophages. BMC, bone marrow chimera

We found that Ly6C^−^ macrophages accounted for the majority of the macrophage population in WT kidneys in CKD after I/R compared to Ly6C^+^ macrophages (Fig. 3a), suggesting they are the major pathogenic subpopulation in the progression of renal fibrosis. Since it was previously reported that the survival of intra-renal Ly6C^−^ macrophages depended on a CX3CL1-CX3CR1 interaction in obstructed kidneys^36^, we stained for CX3CR1 on Ly6C^−^ macrophages after I/R. We found that nearly 90% of Ly6C^−^ macrophages in I/R kidneys expressed CX3CR1 (Fig. 3b), which is regarded as a biomarker of tissue resident macrophages^37^. To further clarify the origin of Ly6C^−^ macrophages, we performed chimerism with the transplantation of GFP-marked bone marrow cells into WT mice (Fig. 3c). The chimerism rate of CD45 is nearly 90% (Supplementary Fig. 2), demonstrating successful chimera formation in which almost all CD45-positive cells derived from bone marrow were GFP^+^ cells. In addition, we found that nearly 95% of Ly6C^−^ monocytes in peripheral blood and 75% of Ly6C^−^ macrophages in kidneys at days 10 and 20 after I/R were GFP-positive (Fig. 3d), indicating that Ly6C^−^ macrophages in injured kidneys were mostly derived from bone marrow. Moreover, we found that this type of GFP^+^Ly6C^−^ macrophage did not express myofibroblast biomarker α-SMA (Fig. 3e), suggesting that they were unable to directly differentiate into myofibroblasts. Altogether, Ly6C^−^ macrophages were the dominant intra-renal macrophages after I/R injury, especially at the chronic phase and most were derived from the bone marrow and did not differentiate into myofibroblasts, indicating an indirect mechanism of Ly6C^−^ macrophages in the progression of renal fibrosis.

### Depletion of renal Ly6C^−^ macrophages in CCR2-deficient mice mitigates AKI-induced fibrosis

**Fig. 4:**
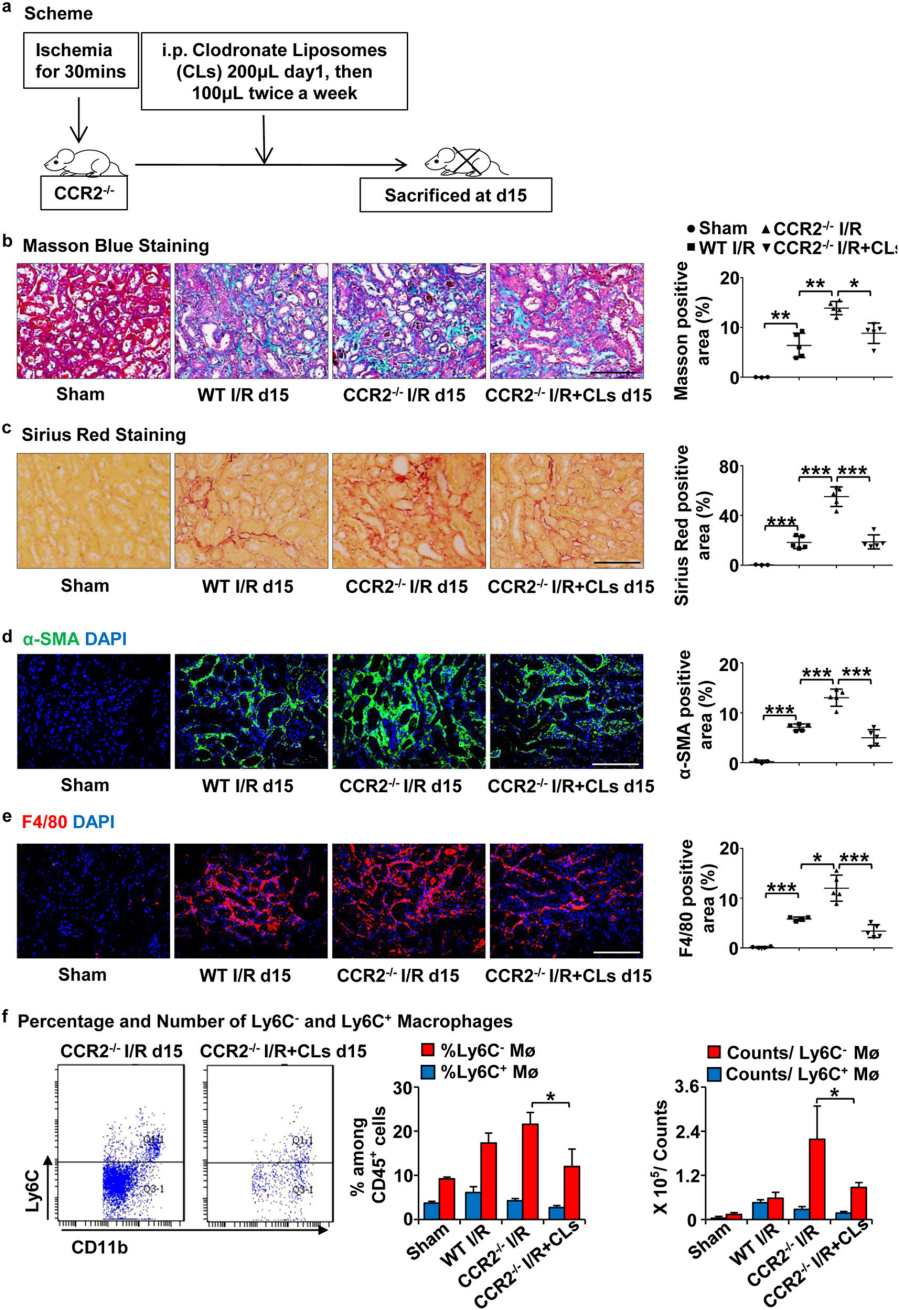
**Depletion of renal Ly6C**^**−**^**macrophages in CCR2 deficient mice mitigates AKI-induced fibrosis.** **a** Scheme. CCR2^−^^/−^ mice were treated with Chlodronate Liposomes (CLs) by i.p. injection 100 μL twice a week after I/R and killed at day 15 (*n* = 5/group). **b**, **c** Representative photomicrographs of Masson Blue staining (**b**) and Sirius red staining (**c**). Corresponding graphs indicate percentage of Masson blue (**b**) and Sirius red (**c**). **d**, **e** Representative photomicrographs for immunofluorescence labeled **d** α-SMA (green) and **e** F4/80 (red). Corresponding graphs indicate the expression of α-SMA (**d**) and F4/80 (**e**). **f** The analyses by flow cytometry of the percentage (middle) and number (right) of Ly6C^−^ and Ly6C^+^ macrophages from the whole kidneys. Graphs and representative plots. Scale bars, 100 μm. *N* = 4–6/group. n.s. > 0.05, *P < 0.05, **P < 0.01, ***P < 0.001. Values were means±SD. Mø, macrophages

To evaluate the role of Ly6C^−^ macrophages in the progression of renal interstitial fibrosis, we treated CCR2^−/−^ mice, which had fewer Ly6C^+^ macrophages in the kidneys, with Clodronate Liposomes (CLs) to deplete Ly6C^−^ macrophages (Fig. 4a). Masson Blue and Sirius Red staining (Fig. 4b, c), and immunofluorescence staining for the expression of α-SMA (Fig. 4d) showed renal fibrosis was alleviated in CCR2^−^^/−^ mice treated with CLs compared to PBS-treated controls. The greater number of macrophages in CCR2^−^^/−^ kidneys at day 15 after I/R were inhibited by CLs injection (Fig. 4e) which correlated with a reduced number of Ly6C^−^ macrophages rather than Ly6C^+^ macrophages (Fig. 4f), suggesting that depleting Ly6C^−^ macrophages mitigated AKI-induced fibrosis in CCR2^−^^/−^ mice.

### Depletion of renal Ly6C^−^ macrophages in WT mice mitigates ischemia-induced AKI and the subsequent renal fibrosis

**Fig. 5:**
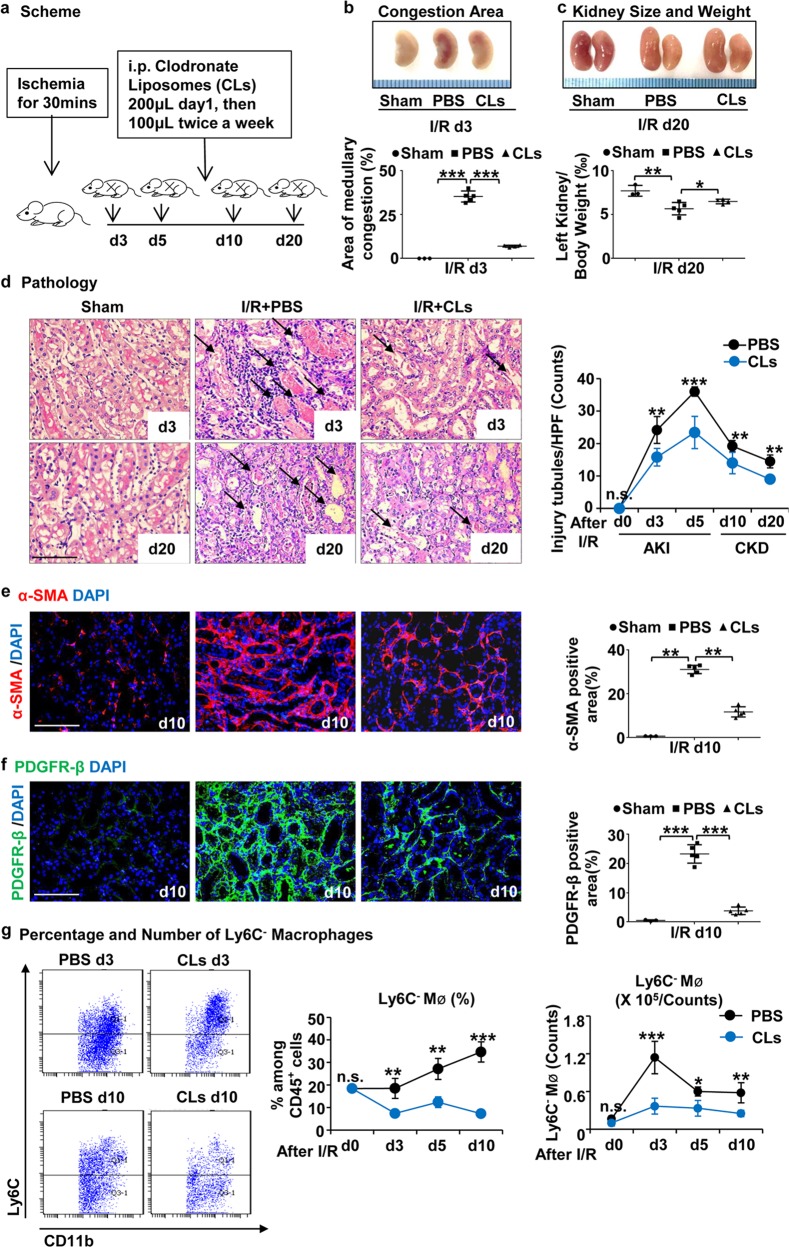
**Depletion of renal Ly6C**^**−**^**macrophages in WT mice by Chlodronate Liposomes mitigates ischemia-induced AKI and the subsequent renal fibrosis.** **a** Scheme. Chlodronate Liposomes (CLs) were injected intraperitoneally 200 μL first day after I/R, and followed by 100 μL twice a week. **b**, **c** Representative kidneys from CLs treated and PBS treated groups for the area of medullary congestion (**b**) and kidney weight (**c**) after I/R. **d** Representative photomicrographs of Periodic Acid-Schiff staining after I/R injury. Graphs indicate the tubular damage after I/R. **e**, **f** Representative photomicrographs for immunofluorescence labeled **e** α-SMA (red) and **f** PDGFR-β (green). Corresponding graphs indicate the expression of α-SMA (**e**) and PDGFR-β (**f**). **g** The percentage (middle) and number (right) of Ly6C^−^ macrophage analyses by flow cytometry from the whole kidneys. Graphs and representative plots. Scale bars, 100 μm. *N* = 4–6/group. n.s. *P* *>* 0.05, *P < 0.05, **P < 0.01, ***P < 0.001. Values were means±SD. Mø, macrophages

In order to verify whether Ly6C^−^ macrophages have the same effect in WT mice, we treated WT mice with CLs (Fig. 5a). We found that the area of medullary congestion was greatly lower in the CLs treatment group compared to PBS (Fig. 5b). Meanwhile, the decrease in kidney weight and size was partially blunted by CLs treatment vs PBS (Fig. 5c), indicating that Ly6C^−^ macrophage depletion alleviated renal injury and the sequential fibrosis. PAS staining further confirmed the attenuated renal injury (Fig. 5d). Immunofluorescence for α-SMA and PDGFR-β (Fig. 5e, f) also showed a reduction in fibrosis. Consistent with previous results, flow cytometry showed that CLs depleted nearly 70% of Ly6C^−^ macrophages (Fig. 5g) and a small proportion of Ly6C^+^ macrophages (Supplementary Fig. 3a). Collectively, these findings suggest that depleting Ly6C^−^ macrophages alleviated not only the ischemia-induced AKI, but also the subsequent renal fibrosis.

### Adoptive transfer of renal Ly6C^−^ macrophages is sufficient to induce renal fibrosis

**Fig. 6:**
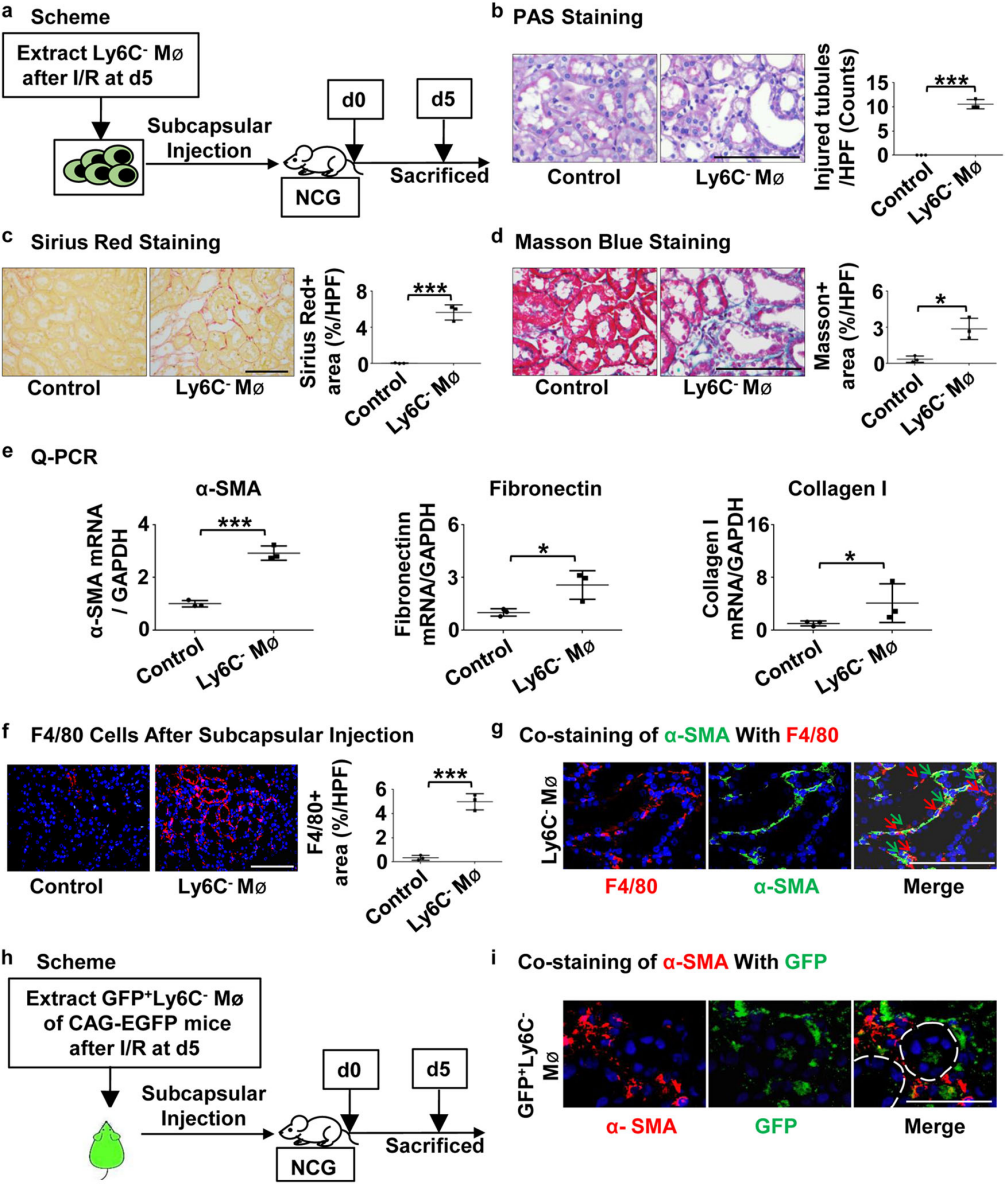
**Adoptive transfer of renal Ly6C**^**−**^**macrophages into immune-deficient kidneys directly induces renal fibrosis.** **a** Scheme. Ly6C^−^ macrophages from I/R kidneys (d5) were injected to NCG mice under renal capsule. The mice were killed at day 5 after injection. Macrophages extracted from sham group as control. **b**–**d** Representative photomicrographs and graphs for Periodic Acid-Schiff staining (**b**), Sirius Red staining (**c**) and Masson Blue staining (**d**). **e** Levels of mRNA encoding α-SMA, Fibronectin, Collagen I by Q-PCR. **f** Representative photomicrographs and graphs for immunofluorescence labeled F4/80 (red). **g** Representative images and double positive analyses for immunofluorescence labeled α-SMA (green) and F4/80 (red). **h** Scheme. Ly6C^−^ macrophages from CAG-EGFP I/R kidneys (d5) were injected to NCG mice under renal capsule. The mice were killed on day 5 after injection. **i** Representative images and double-positive analyses for immunofluorescence labeled α-SMA (red) and GFP (green). Scale bars, 100 μm. *N* = 3/group. *P < 0.05, ***P < 0.001. Values were means ± SD. Mø, macrophages. NCG, NOD-Prkdc^em26Cd52^Il2rg^em26Cd22^/Nju. Mø, macrophages

To further verify the function of Ly6C^−^ macrophages, we extracted Ly6C^−^ macrophages from WT mice at day 5 after I/R, and adoptively transferred them into immune-deficient mice (NOD-Prkdc^em26Cd52^Il2rg^em26Cd22^/Nju, NCG) by subcapsular injection (Fig. 6a). Those intrarenal Ly6C^−^ macrophages at day 5 after I/R were mainly derived from bone marrow (Supplementary Fig. 4). We observed that at day 5 after injection spontaneous renal injury and fibrosis occurred in kidneys injected with Ly6C^−^ macrophages. PAS staining displayed tubular expansion, while Masson Blue and Sirius Red Staining showed that collagen deposition occurred in renal interstitium (Fig. 6b–d). The greater fibrosis was confirmed by Q-PCR of the mRNA encoding α-SMA, fibronectin, and Collagen I (Fig. 6e). Immunofluorescence confirmed the greater macrophage infiltration after transfer of Ly6C^−^ macrophages (Fig. 6f), and that increased F4/80^+^ macrophages were in close proximity to α-SMA^+^ myofibroblasts (Fig. 6g).

In order to further explore whether the Ly6C^−^ macrophages have the ability to directly differentiate into myofibroblasts, we employed subcapsular injection of Ly6C^−^ macrophages from CAG-EGFP mice after I/R injury at day 5 into NCG mice (Fig. 6h). We found that GFP^+^ Ly6C^−^ macrophages appeared in the injured area where α-SMA^+^ myofibroblasts were located (Fig. 6i), but they were not co-localized, suggesting the Ly6C^−^ macrophages-induced fibrosis did not depend on macrophage-myofibroblast transition. Meanwhile, adoptive transfer of renal Ly6C^−^ macrophages into WT kidneys also displayed the same pro-fibrotic effect (Supplementary Fig. 5a–f). Thus, bone marrow derived Ly6C^−^ macrophages mediate ischemia-induced tubular injury and subsequent renal fibrosis via a non-macrophage-myofibroblast transition dependent way.

### Transcriptome analysis of intrarenal Ly6C^−^ macrophages

**Fig. 7:**
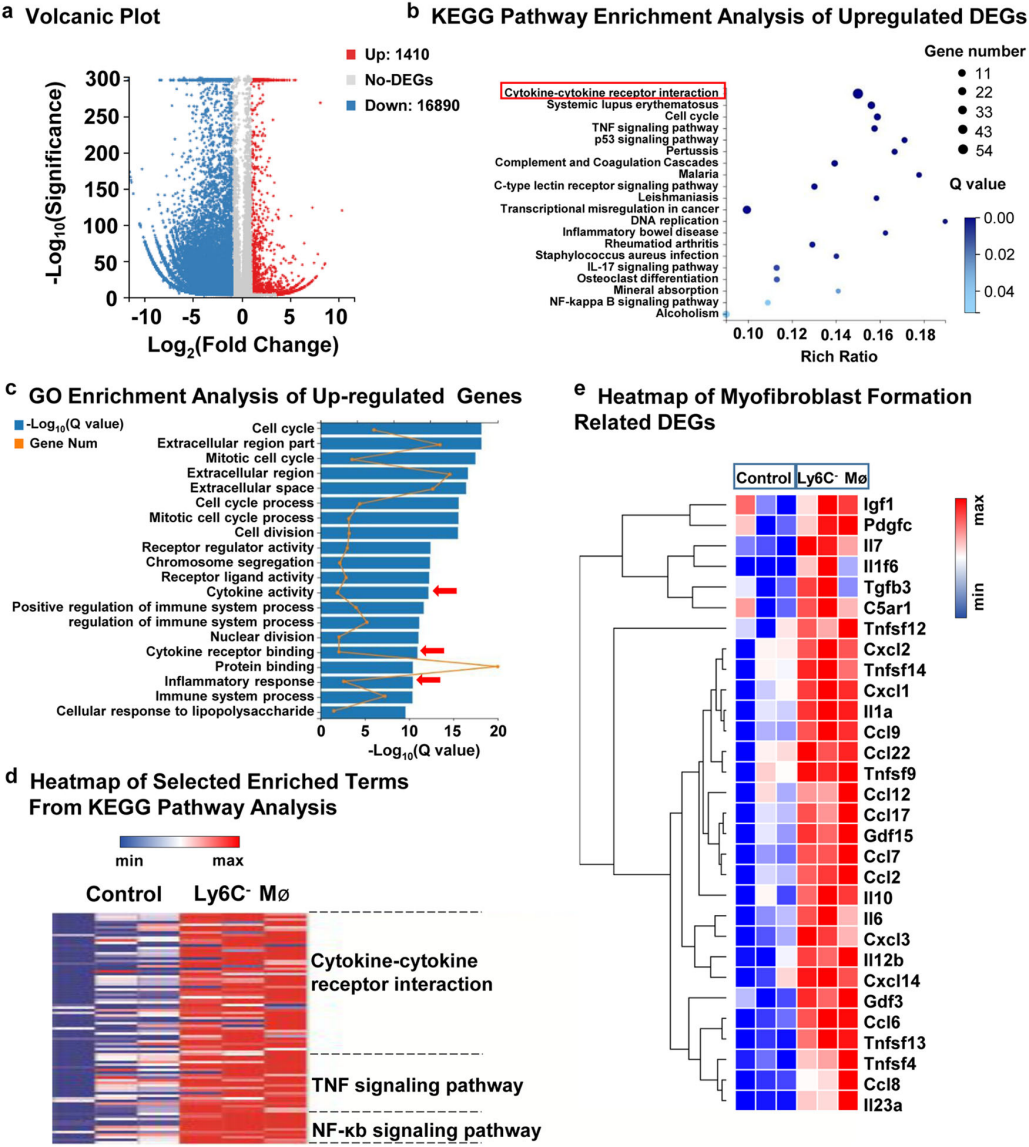
**Transcriptome sequencing of intrarenal Ly6C**^**−**^**macrophages reveals that Ly6C**^**−**^**macrophages secrete profibrotic cytokines and growth factors to promote the transdifferentiation of fibroblasts into myofibroblasts.** Ly6C^−^ macrophages were isolated from kidneys after I/R at day 5 compared with macrophages from kidneys in sham group. **a** Volcano plot of gene expression changes. 1410 upregulated DEGs and 16,890 downregulated DEGs with Fold Change ≥2 and adjusted *P* value ≤0.001 were found. **b** Pathway functional enrichment of all the upregulated DEGs from KEGG pathway analysis (FDR ≤0.01). **c** GO enrichment analysis of all the upregulated DEGs (FDR ≤0.01). **d** Heatmap of selected enriched terms (FDR ≤0.01) from Kegg pathway analysis of upregulated DEGs. **e** Heatmap of myofibroblast formation related DEGs. *N* = 3/group. DEGs, differentially expressed genes. FDR, false discovery rate. KEGG, Kyoto Encyclopedia of Genes and Genomes. GO, Gene ontology. Mø, macrophages

In order to further confirm the profibrotic role and mechanism of Ly6C^−^ macrophage, we performed transcriptome sequencing of Ly6C^−^ macrophages in comparison with macrophages from the sham group. A total of 1410 differentially expressed genes (DEGs) were upregulated in Ly6C^−^ macrophages (Fig. 7a). Kyoto Encyclopedia of Genes and Genomes (KEGG) pathway analysis of all the upregulated DEGs revealed the activation of cytokine receptor interaction (Fig. 7b). Gene ontology (GO) analysis of all the upregulated DEGs confirmed the activation of cytokine activity and inflammatory response (Fig. 7c). The heatmap of the inflammation-associated pathway demonstrated the upregulation of inflammatory factors in Ly6C^−^ macrophages (Fig. 7d). In addition, we observed that a marked activation of inflammatory factors (such as Ccl17, Ccl22, Il10, Il1α and Tnf) and growth factors (such as Igf1, Pdgfc, Gdfs and Tgfb) in Ly6C^−^ macrophages were closely related to the transdifferentiation of fibroblasts. Also, complement C5a receptor 1 (C5ar1) was upregulated in Ly6C^−^ macrophages (Fig. 7e). Furthermore, compared to Ly6C^+^ macrophages, Ly6C^−^ macrophages in the acute phase of I/R expressed less repair-associated genes (Mmp8, Mmp9 and Chil1), suggesting a detrimental role of Ly6C^−^ macrophages in the progression to chronic disease following AKI (Supplementary Fig. 6). Altogether, transcriptome sequencing further validate the role of Ly6C^−^ macrophages in promoting renal fibrosis by activating myofibroblasts.

### Renal Ly6C^−^ macrophages activate fibroblasts in vitro

**Fig. 8:**
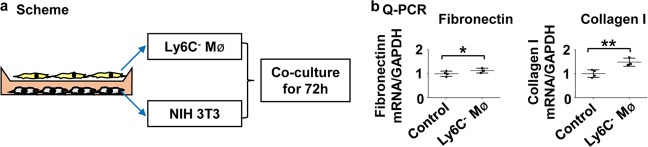
**Renal Ly6C**^**−**^**macrophages directly activate fibroblasts in vitro.** **a** Scheme for the in vitro co-culture of Ly6C^−^ macrophages and fibroblasts. **b** Levels of Fibronectin, Collagen I mRNA by RT-PCR in fibroblasts after co-culture for 72 h. *N* = 3–4/group. n.s. *P* *>* 0.05, *P < 0.05, **P < 0.01. Values were means±SD. Mø, macrophages. NIH 3T3, a cell line of mouse embryonic fibroblast

To clarify the mechanism by which Ly6C^−^ macrophages promoted fibrosis, we co-cultured Ly6C^−^ macrophages from kidneys at day 5 after I/R with NIH 3T3 cells, a cell line of mouse embryonic fibroblast, for 72 h (Fig. 8a). We found that Ly6C^−^ macrophages induced the expression of extracellular matrix proteins (fibronectin and collagen I) from NIH 3T3 cells as measured by Q-PCR of their corresponding mRNA from cellular extracts (Fig. 8b). These results suggest that Ly6C^−^ macrophages assist in the transdifferentiation of fibroblasts into myofibroblasts.

## Discussion

In this study, we revealed that the intra-renal Ly6C^−^ macrophages, which primarily originate from the bone marrow and peripheral circulation, were the most abundant subpopulation after I/R, especially in the chronic phase. These Ly6C^−^ macrophages accelerated kidney injury, activated myofibroblasts and in turn exacerbated the ischemia-induced renal fibrosis in both WT and CCR2-deficient mice. These findings challenge several of the current concepts about ischemia-induced CKD and macrophages. Firstly, although CCR2 deficiency inhibits the recruitment of inflammatory Ly6C^+^ macrophages and improves ischemia-induced AKI, it does not alleviate the subsequent CKD, because of the compensatory replenishment of intra-renal Ly6C^−^ macrophages. Secondly, the cell surface marker Ly6C cannot precisely define kidney resident macrophages under pathological conditions, as most of them are derived from the periphery after I/R. Thirdly, our results provide initial in vivo validation that bone marrow-derived Ly6C^−^ macrophages are a type of toxic macrophages after kidney ischemia injury, which provides a novel target for the treatment of ischemia-induced CKD.

To specifically study Ly6C^−^ macrophages, we used CCR2^−/^^−^ mice, which eliminated the interference of Ly6C^+^ macrophages due to the lack of the chemokine receptor required for recruitment of these monocytes from the bone marrow to inflammatory tissue. Renal damage and inflammatory infiltration in CCR2^−/−^ mice were significantly reduced in the acute phase after I/R injury with decreased intra-renal macrophage infiltration, which is consistent with previous reports^38,39^, though these earlier studies were not extended into the late chronic phase after I/R to confirm the pro-fibrotic or anti-fibrotic effect of CCR2 deficiency in mice. Our data showed a compensatory increase in macrophage infiltration in the CCR2^−/−^ kidneys at the CKD phase after I/R, which provided a reliable indication that the replenished Ly6C^−^ macrophages acted as a hidden culprit to promote the progression of renal fibrosis after I/R.

In previous studies, the macrophage subtypes and their functions were not clearly determined, as there were several limitations in the tools for macrophage depletion^40,41^. Ferenbach et al. demonstrated that macrophage depletion by CLs after I/R improved renal injury, while depletion by diphtheria toxin administration to CD11b-iDTR mice did not. The reason for this discrepancy is derived from the difference in tissue monocyte-lineage of the depleted macrophage subtypes by the above two tools^42,43^. In our study, we found that CLs depleted most of Ly6C^−^ macrophages and a small portion of Ly6C^+^ macrophages, which is consistent with a recent study^44^. However, the relation between these subtypes is still unclear and further studies will be required for more precise depletion of macrophage subtypes.

The origin of Ly6C^−^ macrophages is controversial. Whether the increased Ly6C^−^ macrophages after injury in tissues are derived from circulating Ly6C^+^ monocytes or solely from locally proliferating resident macrophages is not fully understood. Some studies suggested that Ly6C^−^ macrophages self-maintain locally with minimal contribution from circulating monocytes^45,46^, while other reports demonstrated that in the inflammatory microenvironment, Ly6C^–^ macrophages can be replenished by circulating monocytes^22,43,47,48^, To further confirm the proportion of monocyte-derived Ly6C^−^ macrophages, we used bone marrow chimerism. Our results indicated that circulating monocytes were constituted mostly of Ly6C^−^ macrophages (nearly 75%) and that only 25% of those originated from in situ proliferation. This suggests that defining resident renal macrophages by the cell surface marker Ly6C is inappropriate in I/R-induced renal injury.

Under physiological condition, Ly6C^−^ macrophages per se are the kidney-resident macrophages and derived from the embryonic yolk sac^11^. They maintain themselves in adults by self-renewal, and have the function of surveying endothelial integrity^49^. However, by adoptive transfer we found that Ly6C^−^ macrophages from I/R-induced kidney injury are mostly bone marrow derived and profibrotic compared to macrophages extracted from the sham group control. As such, our results suggest bone marrow-derived Ly6C^−^ macrophages play a different role from resident Ly6C^−^ macrophages in the AKI-to-CKD transition.

In this study we did not detect the differentiation of Ly6C^−^ macrophages into myofibroblasts, which has been proposed as the major mechanism for macrophage-mediated renal fibrosis^50,51^. Thus, we explored whether macrophages secrete certain cytokines to affect the renal microenvironment. It was reported that Ly6C^−^ kidney macrophages expressed relatively high levels of Ccl17, Ccl22, Igf-1, and Pdgfβ^22^, indicating that they have the capacity to promote fibrosis by paracrine signaling. Regarding the indirect mechanism of Ly6C^−^ macrophages in promoting renal fibrosis, we performed RNA sequencing of Ly6C^−^ macrophages, which showed that Ly6C^−^ macrophages secrete various cytokines and growth factors to potentially promote the transdifferentiation of fibroblasts into myofibroblasts. In addition, co-culturing Ly6C^−^ macrophages with NIH 3T3 fibroblasts also confirmed that Ly6C^−^ macrophages induced the expression of extracellular matrix proteins from NIH 3T3 cells. However, further studies are necessary to investigate the mechanisms by which Ly6C^−^ macrophages affect fibroblasts.

Recently, Thorenz et al. identified that complement activation plays an important role in the progression of AKI-CKD^35^. In our study, we observed that CCR2^−/−^ kidneys showed a higher level of complement activation in the CKD phase compared to WT mice. In addition, RNA sequencing also showed relatively high expression of C5ar1 in Ly6C^−^ macrophages. These results suggest that complement activation related to Ly6C^−^ macrophages may also contribute to the continuous progression of AKI.

In conclusion, this study provides evidence that bone marrow-derived Ly6C^−^ macrophages directly induce renal injury and fibrosis after I/R injury. Their profibrotic effect is achieved through paracrine signaling in fibroblasts, activating them into myofibroblasts and in turn worsens the ischemia-induced renal fibrosis. These biological insights will aid in the development or improvement of existing therapies for the treatment of ischemia-induced chronic kidney disease.

## Supplementary information

Supplementary Figure 1

Supplementary Figure 2

Supplementary Figure 3

Supplementary Figure 4

Supplementary Figure 5

Supplementary Figure 6
