# Supplementary figures and images for: Bone marrow-derived Ly6C− macrophages promote ischemia-induced chronic kidney disease
Source: Cell Death Dis. 2019 Mar 29;10(4):291. doi: 10.1038/s41419-019-1531-3 (PMC6440948; doi:10.1038/s41419-019-1531-3)

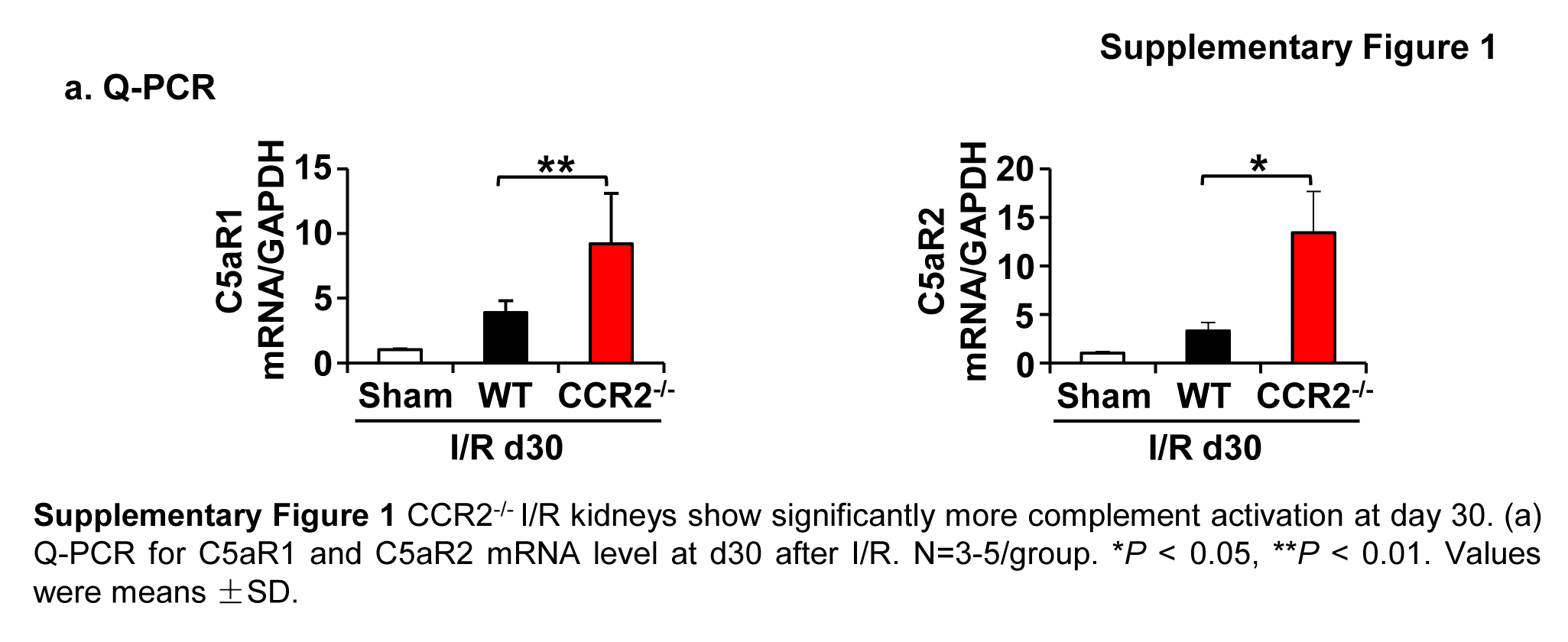

Supplement: Supplementary file 1 — Supplementary Figure 1 [file 41419_2019_1531_MOESM1_ESM.jpg]

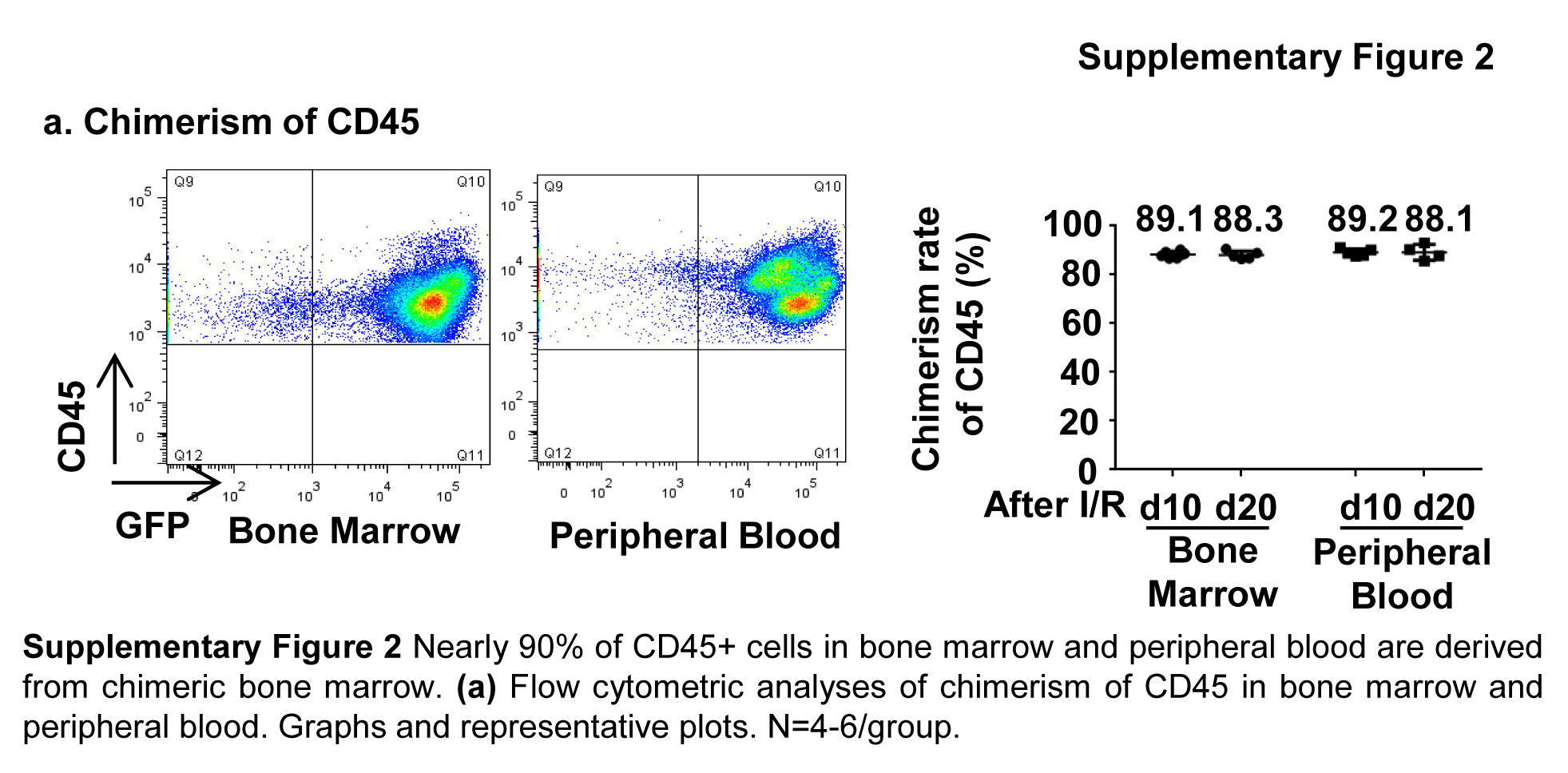

Supplement: Supplementary file 2 — Supplementary Figure 2 [file 41419_2019_1531_MOESM2_ESM.jpg]

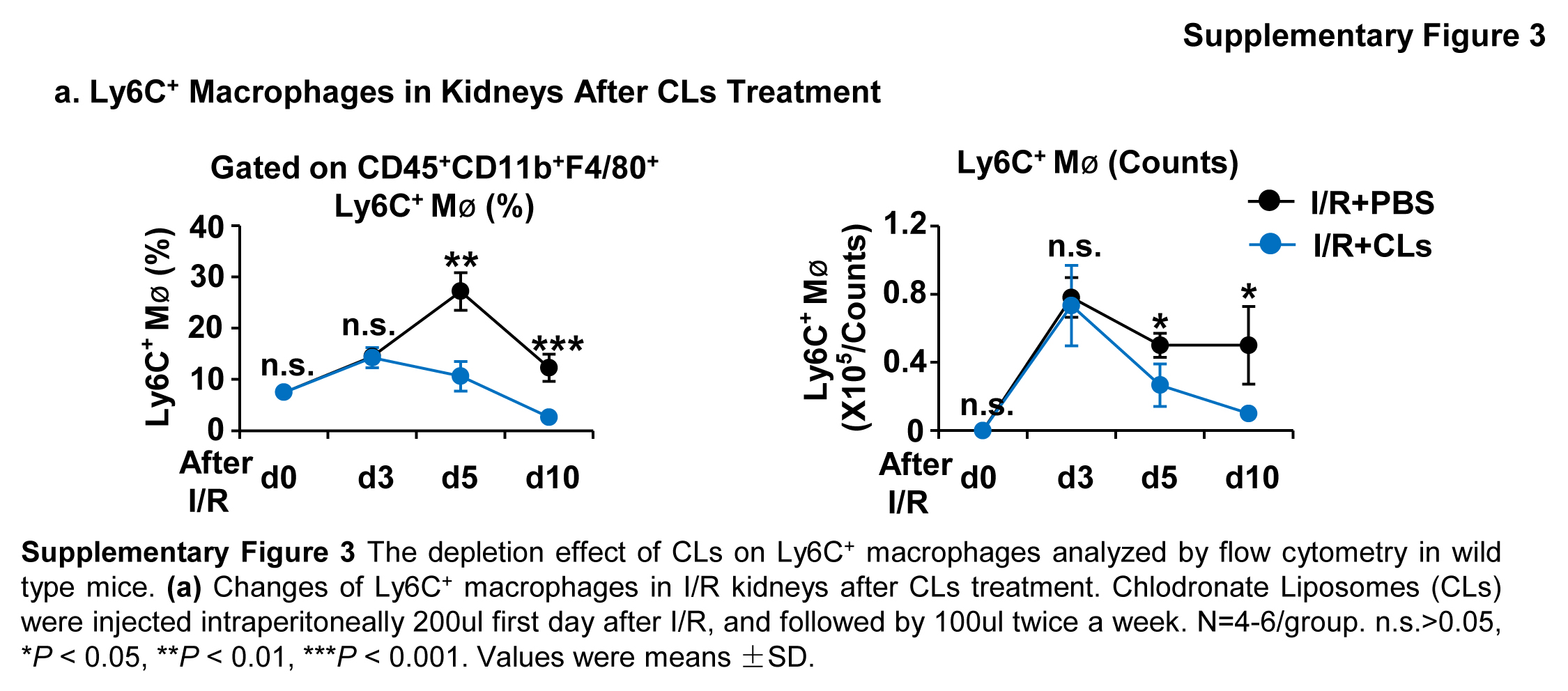

Supplement: Supplementary file 3 — Supplementary Figure 3 [file 41419_2019_1531_MOESM3_ESM.jpg]

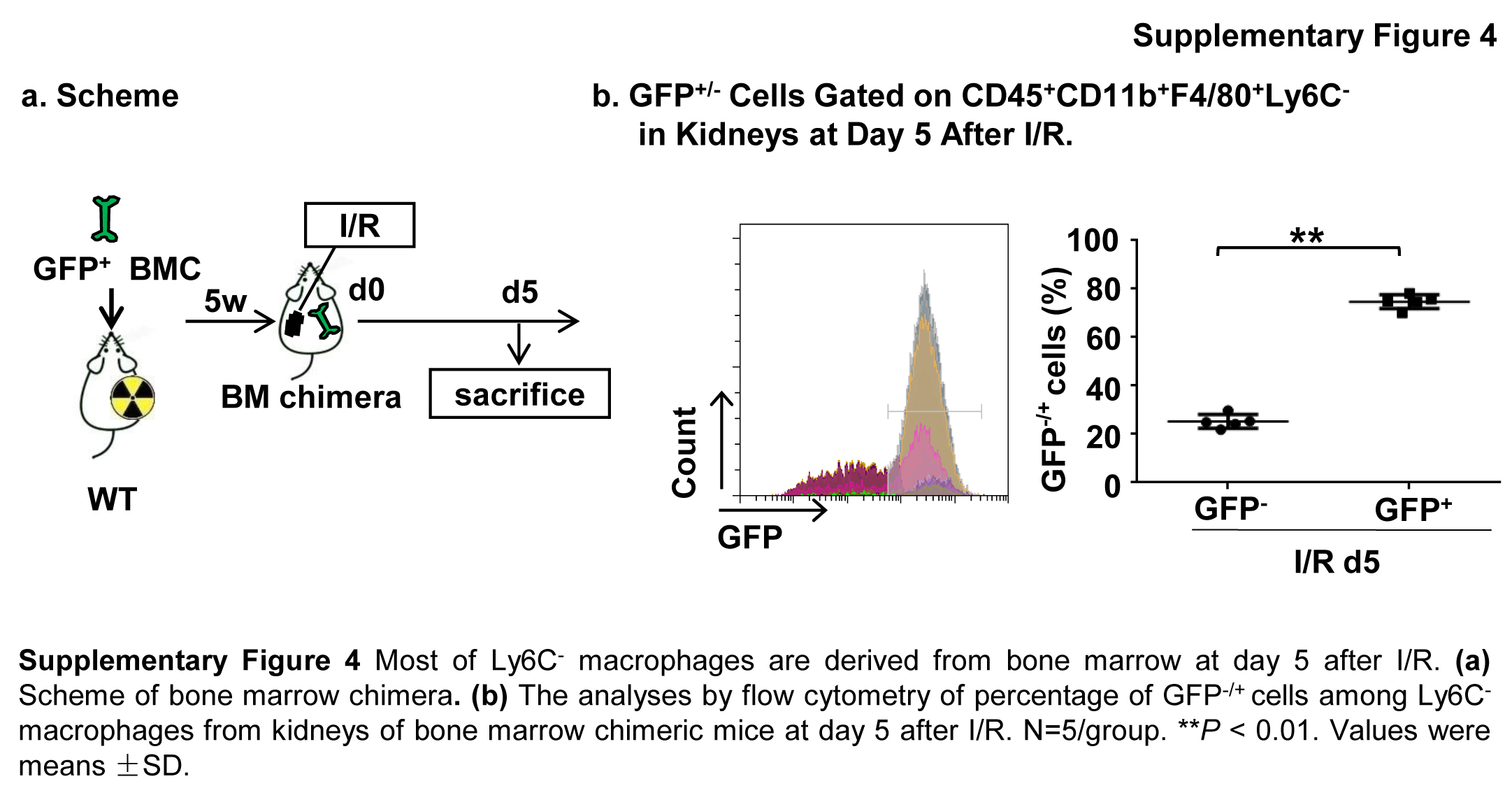

Supplement: Supplementary file 4 — Supplementary Figure 4 [file 41419_2019_1531_MOESM4_ESM.jpg]

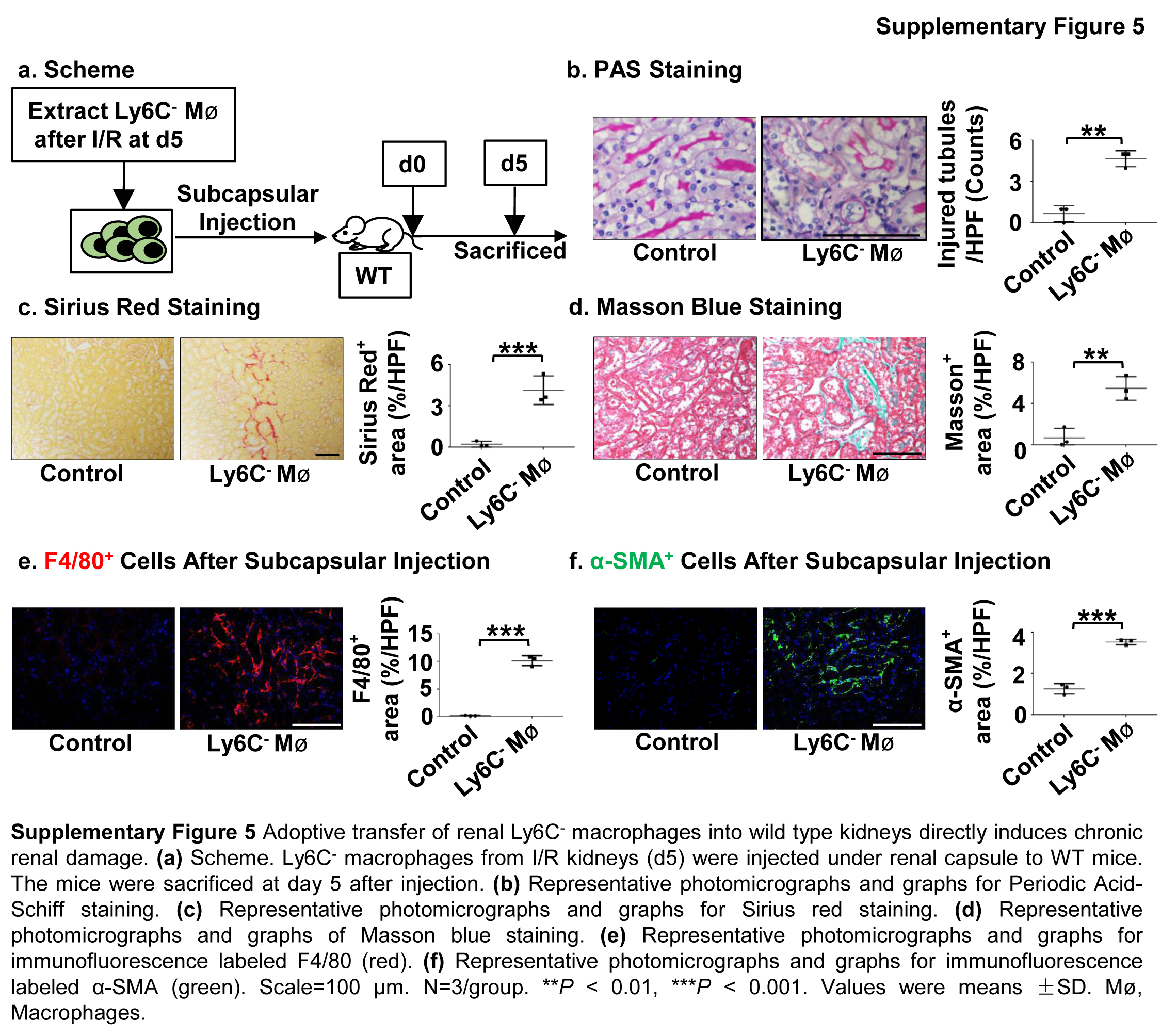

Supplement: Supplementary file 5 — Supplementary Figure 5 [file 41419_2019_1531_MOESM5_ESM.jpg]

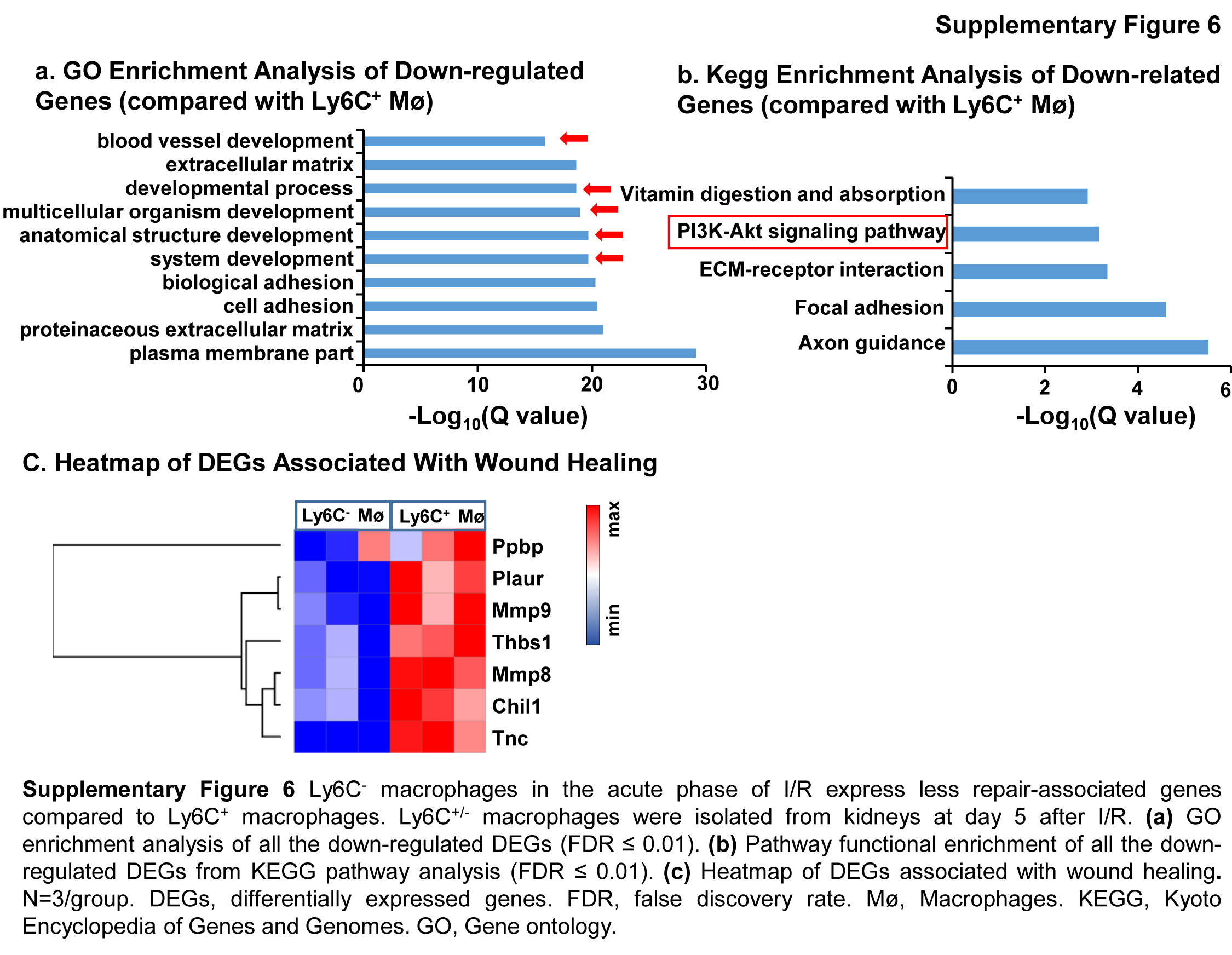

Supplement: Supplementary file 6 — Supplementary Figure 6 [file 41419_2019_1531_MOESM6_ESM.jpg]
